# BRCA1 expression modulates chemosensitivity of BRCA1-defective HCC1937 human breast cancer cells

**DOI:** 10.1038/sj.bjc.6600859

**Published:** 2003-04-15

**Authors:** P Tassone, P Tagliaferri, A Perricelli, S Blotta, B Quaresima, M L Martelli, A Goel, V Barbieri, F Costanzo, C R Boland, S Venuta

**Affiliations:** 1 Oncology Unit, Department of Experimental and Clinical Medicine, ‘Magna Graecia’ University, 88100 Catanzaro, Italy; 2 Department of Medicine and Comprehensive Cancer Center, University of California, San Diego, La Jolla, CA 92093-0688, USA

**Keywords:** BRCA1, BRCA1-defective cells, hereditary tumours, breast hereditary cancer, cisplatin, doxorubicin, paclitaxel

## Abstract

Germline mutations of the tumour suppressor gene BRCA1 are involved in the predisposition and development of breast cancer and account for 20–45% of all hereditary cases. There is an increasing evidence that these tumours are characterised by a specific phenotype and pattern of gene expression. We have hypothesised that differences in chemosensitivity might parallel molecular heterogeneity of hereditary and sporadic breast tumours. To this end, we have investigated the chemosensitivity of the BRCA1-defective HCC1937 breast cancer cell line, and the BRCA1-competent MCF-7 (hormone-sensitive) and MDA-MB231 (hormone-insensitive) breast cancer cell lines using the MTT assay. The 50% inhibitory concentration (IC_50_) for the individual compounds were derived by interpolate plot analysis of the logarithmic scalar concentration curve after a 48 h exposure. HCC1937 cells were significantly (*P*<0.005) more sensitive to cisplatin (CDDP) (IC_50_ : 30–40 *μ*M) compared with MCF-7 (IC_50_ : 60–70 *μ*M) and MDA-MB231 (IC_50_ : 90–100 *μ*M) cells. On the other hand, BRCA1-defective breast cancer cells were significantly less sensitive to doxorubicin (Dox) (IC_50_ : 45–50 *μ*M) compared with MCF-7 (IC_50_ : 1–5 *μ*M) and MDA-MB231 (IC_50_ : 5–10 *μ*M) (*P*<0.02), as well as to paclitaxel (Tax) (IC_50_ : >2 *μ*M for HCC1937, 0.1–0.2 *μ*M for MCF-7 and 0.01–0.02 *μ*M for MDA-MB231) (*P*<0.001). Full-length BRCA1 cDNA transfection of BRCA1-defective HCC1937 cells led to the reconstituted expression of BRCA1 protein in HCC1937/^WT^BRCA1-derived cell clone, but did not reduce tumour cell growth in soft agar. BRCA1 reconstitution reverted the hypersensitivity to CDDP (*P*<0.02), and restored the sensitivity to Dox (*P*<0.05) and Tax (*P*<0.001), compared with parental HCC1937 cells. Taken together, our findings suggest a specific chemosensitivity profile of BRCA1-defective cells *in vitro*, which is dependent on BRCA1 protein expression, and suggest prospective preclinical and clinical investigation for the development of tailored therapeutical approaches in this setting.

Breast carcinoma is the most important malignant disease for Western women. An hereditary form has been identified, which is related to inherited cancer-predisposing germline mutations. Germline mutations of BRCA1 gene have been identified in 15–20% of women with a family history of breast cancer and 60–80% with family history of both breast and ovarian cancer (Nathanson *et al*, 2001).

The human BRCA1 gene, which was mapped in 1990 and was subsequently cloned in 1994 (Miki *et al*, 1994), is located at 17q21, and encodes a nuclear phosphoprotein of 1863 amino acids that contains several functional domains (Chen *et al*, 1996). These domains interact with numerous molecules, including DNA damage-repair proteins and other proteins with a regulatory role in fundamental cellular processes (Deng and Brodie, 2000). In fact, BRCA1 participates in a number of functions, including the regulation of cell proliferation and in the events that require chromatin remodelling (Hu *et al*, 1999; Bochar *et al*, 2000). Overexpression of the BRCA1 gene interferes with breast cancer cell growth *in vitro* (Holt *et al*, 1996) and may induce apoptosis if overexpressed in transfected cancer cells (Shao *et al*, 1996), supporting the hypothesis that BRCA1 is a tumour suppressor gene.

Recent reports have highlighted the role of BRCA1 in maintaining the genomic integrity and regulating sensitivity to DNA-damaging agents. BRCA1 becomes activated by phosphorylation and localises after exposure to DNA double-strand break damage (Scully *et al*, 1997a). It interacts with Rad51 and BRCA2 in subnuclear foci, and activates the recombination repair process (Scully *et al*, 1997b; Chen *et al*, 1999). Using isogenic mouse embryonic stem cell lines, it has been demonstrated that BRCA1 is required for the formation of subnuclear Rad51 complexes in response to cellular damage by ionising radiation or cisplatin (CDDP) treatment, suggesting that BRCA1 contributes to damage repair and/or tolerance by promoting assembly of the Rad51 complex (Bhattacharyya *et al*, 2000). It has been shown that upregulation of BRCA1 expression leads to increased resistance to CDDP in ovarian cancer cells (Husain *et al*, 1998). It has also been shown that restoration of BRCA1 expression in the BRCA1-defective HCC1937 human breast cancer cell line induces radioresistance (Abbott *et al*, 1999). Anti-BRCA1 ribozymes led to increased sensitivity to the DNA-damaging agents CDDP and etoposide, and to resistance to the microtubule-interfering agents paclitaxel (Tax) and vincristine (Lafarge *et al*, 2001). In this cell system, the molecular mechanism of resistance to microtubule-interfering agents was correlated to transcriptional changes in genes involved in the c-Jun NH(2)-terminal kinase (JNK) pathway. More recently, it has been reported that the expression of a truncated BRCA1 mutant with a dominant-negative activity modifies chemosensitivity in a mouse ovarian cancer cell line (Sylvain *et al*, 2002). Taken together, these findings suggest that changes in the sensitivity to antitumor agents could indeed be a part of the phenotype of breast carcinomas arising in the hereditary setting and could be of relevance in the treatment of these tumours.

We investigated the effects of BRCA1 inactivation on drug sensitivity in human breast cancer cell lines. We asked whether human breast cancer cells derived from an individual harbouring a BRCA1 germline mutation and complete loss of BRCA1 function (BRCA^−/−^) would have a specific chemosensitivity profile compared to the sporadic tumour-derived hormone-sensitive MCF-7 and hormone-insensitive MDA-MB231 cell lines. We reasoned that BRCA^−/−^ breast cancer cells offer the specific molecular pathology milieu for experimentally addressing the issues relevant to the management of breast tumours in the hereditary setting. The specific role of BRCA1 in determining drug sensitivity was also investigated by comparative analysis of parental HCC1937 with HCC1937/^WT^BRCA1-derived cell line with reconstitued BRCA1 protein expression after BRCA1 full-length cDNA transfection.

## MATERIALS AND METHODS

### Cell cultures and reagents

HCC1937, MCF-7 and MDA-MB231 cell lines were purchased from the American Type Culture Collection (Rockville, MD, USA). HCC1937 cells were grown in RPMI 1640 medium (Life Technologies, Paisley, UK), while MCF-7 and MDA-MB231 were grown in Dulbecco's modified Eagle's medium (DMEM) (Life Technologies). All media were supplemented with 10% fetal bovine serum (FBS), 2 mM L-glutamine, 100 *μ*g ml^−1^ streptomycin and 100 U ml^−1^ penicillin. All cell lines were cultured at a constant temperature of 37°C in a 5% carbon dioxide (CO_2_) humidified atmosphere. HCC1937 transfected with full-length BRCA1 cDNA cells (HCC1937/^WT^BRCA1) were generated in this laboratory. CDDP and Tax were purchased from Bristol-Myers Squibb (NJ, USA). Doxorubicin (Dox) was purchased from Pharmacia & Upjohn (Dublin, Ireland).

### Drug sensitivity assays

Cell proliferation analysis was performed on breast cancer cells in the presence of increasing concentrations of antitumour drugs by the thiazolyl blue (MTT) assay. Briefly, breast cancer cells (1 × 10^4^ well^−1^) were plated in 96-well plates. At 24 h after the initial seeding of the cells, various drug treatments were carried out for 48 h. Subsequently, the wells were incubated with 10 *μ*l well^−1^ of MTT at 5 mg ml^−1^ (Sigma Chemical Co., St Louis, MO, USA) for 1 h at 37°C. Then 100 *μ*l of 0.04 N HCl in isopropanol was added and the absorbance was measured in a microplate reader at a wavelength of 620 nm. A value of 100% was assigned to untreated control cultures, and the concentration of drug that reduced the number of viable cells to 50% after 48 h of exposure (IC_50_) was derived by an interpolate logaritmic concentration curve. Results were derived from at least three independent sets of triplicate experiments.

### cDNA transfection

Parental HCC1937 were transfected with a pcDNA 3.1 plasmid containing the full-length BRCA1 gene. Stable transfectants were selected and used for drug sensitivity assays. For stable transfections, cells at 30–40% confluency were incubated overnight with 2 *μ*g of plasmid DNA, using the FuGENE 6 transfection reagent (Roche Molecular Biochemicals, Monza, Italy) according to the manufacturer's instructions. Cells were then selected in G418 (0.4 mg ml^−1^) (Invitrogen Life Technologies, La Jolla, CA, USA). Cell clones that stably expressed G418 and retained growth potential were assayed for BRCA1 expression by Western blot assay.

### Western Blot analysis of BRCA1 protein

Protein extracts were prepared as described elsewhere (Faniello *et al*, 1999) and equal amounts (50 *μ*g) were resolved by SDS–polyacrylamide gel electrophoresis and transferred to nitrocellulose membranes (Hybond ECL Nitrocellulose Membrane, Amersham Pharmacia Biotech, Piscataway, NJ, USA). After addition of the blocking mixture, the membrane was incubated with a 1 : 200 dilution of BRCA1 D9 mouse monoclonal IgG2a antibody for 2 h at room temperature. Bound antibody was detected using a 1 : 2000 dilution of HRP-conjugated goat anti-mouse IgG antibody for 1 h at room temperature. All the antibodies were purchased from Santa Cruz Biotechnology (Santa Cruz, CA, USA). The signal was detected using ECL (Santa Cruz Biotechnology, Santa Cruz, CA, USA).

### Soft agar growth assay

Soft agar assay was performed using 5 × 10^4^ HCC1937 parental cells, mock-transfected HCC1937 cells and HCC1937/^WT^BRCA1 cells, assayed for BRCA1 protein expression. They were suspended in 1.5 ml of 3% Noble Agar (Difco, Kansas City, MO, USA), 100 mM triptose phosphate buffer (Difco, USA), 10% FBS RPMI 1640 medium and layered on 7 ml of a similar buffer with Noble Agar concentration increased to 5% in 60 mm culture dishes. Plates were incubated at a constant temperature of 37°C with a humidified atmosphere of 5% CO_2_ for 4 weeks, stained with crystal violet and scored for colonies that were greater than 60 *μ*m.

### Flow cytometric analysis of apoptosis

Apoptotic cell death was analysed by the Annexin-V kit (MedSystems Diagnostics, Vienna, Austria). Briefly, cells were incubated with Annexin-V-FITC in binding buffer (provided by the manufacturer) for 10 min at room temperature, washed and resuspended in the same buffer as described by the manufacturer. Analysis of apoptosis was performed by flow cytometry (FACScan, Becton Dickinson, San Josè, CA, USA).

### Statistical analysis

Results are expressed as mean±s.d. The statistical significance of differences between the experimental groups was analysed using the *t*-test; differences were considered significant when *P*≤0.05.

## RESULTS

### Differential sensitivity of HCC1937, MCF-7 and MDA-MB231 cell lines to cytotoxic drugs

We examined the differences in the sensitivity of the HCC1937 human breast cancer cell line harbouring a BRCA1 mutation and with complete loss of BRCA1 function, compared with hormone-sensitive MCF-7 and hormone-insensitive MDA-MB231 cell lines, to chemotherapeutic agents that are commonly used in the treatment of breast cancer.

**Figure 1:**
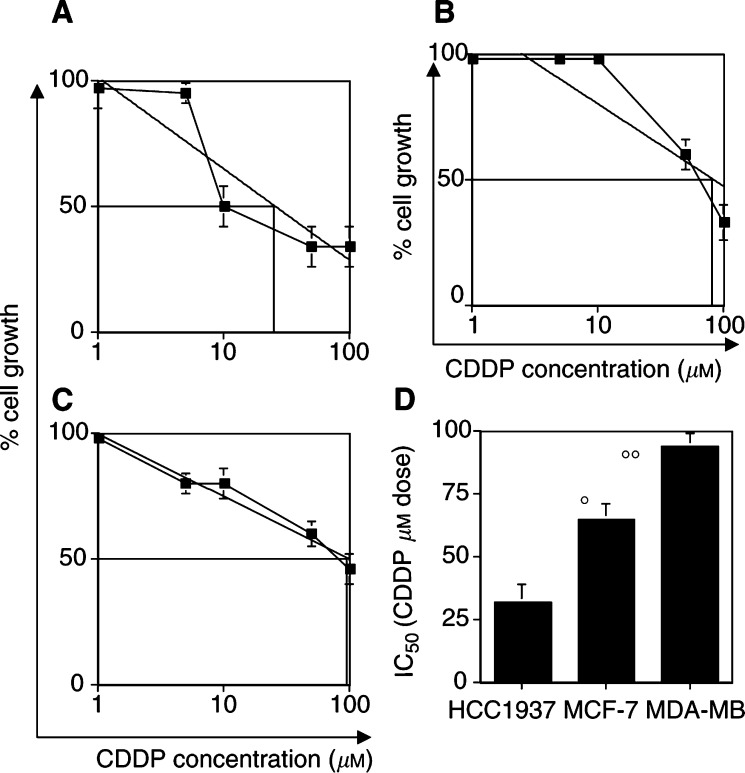
Differential sensitivity among BRCA1-defective and BRCA1-competent cells to CDDP. In (**A**) HCC1937 cells, (**B**) MCF-7 cells and (**C**) MDA-MB231 cells, the dose-related activity of CDDP is shown as tumour cell growth. Analysis was performed after 48 h exposure to the drug and the IC_50_ was calculated by interpolate logarithmic curve. In (**D**), the value of IC_50_ determined for each cell line is shown. **°** and **°°**: *P*<0.005.

Treatment with the DNA alkylating and crosslinking agent CDDP (1–100 *μ*M) produced a dose-dependent reduction in cell growth in all cell lines after 48 h of treatment (Figure 1A–C

). However, the HCC1937 (BRCA1-defective) cell line was 2–3-fold more sensitive to CDDP compared with BRCA1-competent cell lines, with an IC_50_ in a range of 30–40 *μ*M drug concentration (Figure 1D). MCF-7 and MDA-MB231 cells showed significantly higher IC_50_'s, which were in the range of 60–70 and 90–100 *μ*M, respectively (Figure 1D).

**Figure 2:**
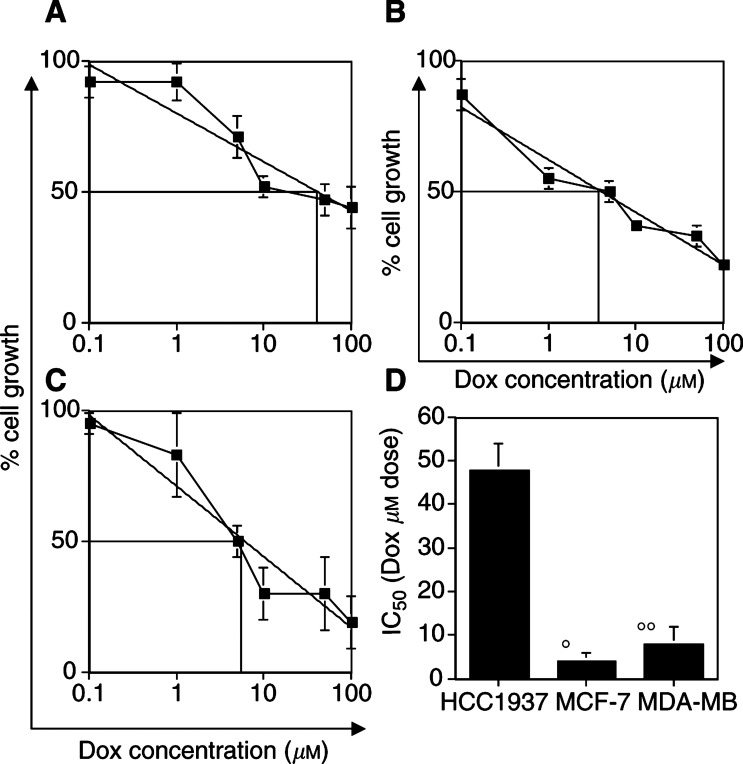
Differential sensitivity among BRCA1-defective and BRCA1-competent cells to Dox. In (**A**) HCC1937 cells, (**B**) MCF-7 cells and (**C**) MDA-MB231 cells, the dose-related activity of Dox is shown. Interpolate logarithmic curve and the IC_50_ of cell growth are shown in each quadrant. In (**D**), the value of IC_50_ determined for each cell line is shown. **°** and **°°**: *P*<0.02.

The treatment of these cells with the DNA helix intercalator and topoisomerase II inhibitor Dox or with the mitotic spindle poison Tax showed an opposite pattern of chemosensitivity compared with CDDP. Increasing doses of Dox (1–100 *μ*M) produced a progressive reduction in cell growth in all cell lines after 48 h (Figure 2A–C

). HCC1937 cells were resistant to Dox, with an IC_50_ in the range of 45–50 *μ*M. Compared with the other cell lines, HCC1937 cells are approx 5–10-fold more resistant (Figure 2D). In fact, the MCF-7 and MDA-MB231 cells showed lower IC_50_ concentrations and were in a range of 1–5 and 5–10 *μ*M, respectively (Figure 2D).

**Figure 3:**
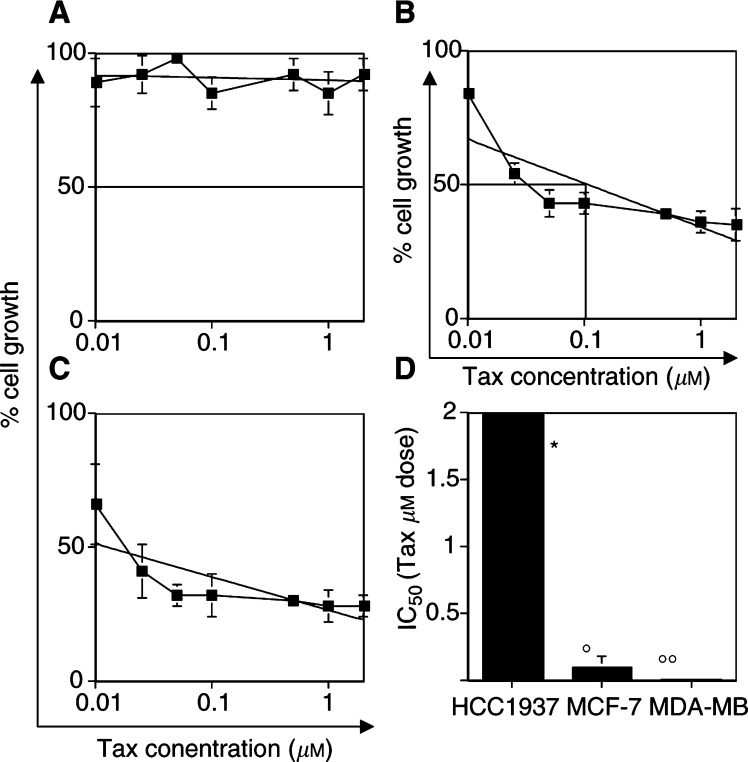
Differential sensitivity among BRCA1-defective and BRCA1-competent cells to Tax. In (**A**) HCC1937 cells, (**B**) MCF-7 cells and (**C**) MDA-MB231 cells, the dose-related activity of Tax is shown. Interpolate logarithmic curve and the IC_50_ of cell growth are shown in each quadrant. In (**D**), the value of IC_50_ determined for each cell line is shown. ^*^: Maximum of concentration used, no IC_50_ was achieved at this dose of drug. **°** and **°°**: *P*<0.001.

We then studied the effect of Tax on BRCA1-defective and BRCA1-competent cancer cells. Tax is an active microtubule-interfering agent for the treatment of advanced breast cancer. Figure 3

shows the differential response of these breast cancer cell lines to Tax-induced cytotoxicity. MCF-7 and MDA-MB231 cells were highly sensitive to Tax (IC_50_: 0.1–0.2 *μ*M and 0.01–0.02 *μ*M, respectively) (Figure 3B and C), whereas the HCC1937 cells showed substantial resistance (Figure 3A). In fact, no IC_50_ concentration was detected at doses inducing growth arrest in the other cell lines even at 20-fold higher doses (2 *μ*M) (Figure 3D). These results indicate that BRCA1-defective breast cancer cells are highly responsive to CDDP treatment but resistant to Dox or Tax, suggesting a differential pattern of chemosensitivity specific for breast cancer cells with loss of BRCA1 function.

### Western blot analysis of BRCA1 expression and clonogenic soft agar growth of stable transfectants HCC1937/^WT^BRCA1

**Figure 4:**
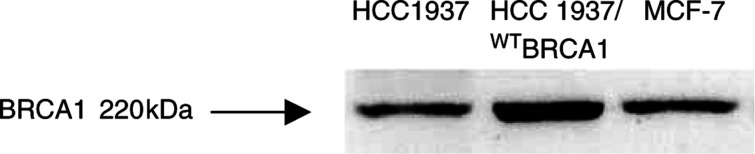
cDNA transfection reconstitutes BRCA1 expression. Western blot analysis of BRCA1 protein expression in HCC1937, HCC-1937/^WT^BRCA1 and MCF-7 cells.

To demonstrate that this chemosensitivity profile is indeed associated with the functional activity of the BRCA1 gene, we generated full-length cDNA transfectants in order to perform comparative chemosensitivity assays and apoptotic analysis between BRCA1-defective and restored cells. By Western blot analysis, we evaluated the restoration of BRCA1 expression in the G418/resistant cDNA/transfectant cells. Figure 4

shows increased expression of BRCA1 protein in transfected cells, suggesting effective restoration of protein expression. Specificity of mAb used for Western blot analysis has been further demonstrated in our laboratory by its capacity of immunoprecipitating BRCA1 protein which was capable of interacting with BASC protein (Quaresima *et al*, manuscript in preparation). The stable transfectant was therefore defined HCC1937/^WT^BRCA1. In order to demonstrate growth potential and maintenance of the malignant phenotype, parental HCC1937, HCC1937/pcDNA3 (empty vector transfected) and HCC1937/^WT^BRCA1 were assayed for clonogenic soft agar growth. No significant changes in the number and dimension of colonies were observed (data not shown). While BRCA1 transfection is generally considered to inhibit breast cancer growth by itself (Holt *et al*, 1996), it is possible to argue that our experimental conditions and selection allows the isolation of cellular clones insensitive to the growth-suppressing effect of a functional BRCA1 gene. We considered that for the purpose of comparative drug sensitivity evaluation, transfectants retaining an analogous growth potential as compared to the unreconstituted counterpart should be necessary. Stable BRCA1 transfectants HCC-1937 have been successfully generated by the use of wild-type gene inserted into different vectors (Ruffner *et al*, 2001; Andrews *et al*, 2002). Moderate increase of BRCA1 expression after transfection of a full-lenght construct under a Tet-inducible promoter did not affect the growth of BRCA1 wild-type MDA MB 435 (Mullan *et al*, 2001).

### Full-length transfection of BRCA1 in HCC1937 induces resistance to CDDP and restores sensitivity to Dox and Tax

**Figure 5:**
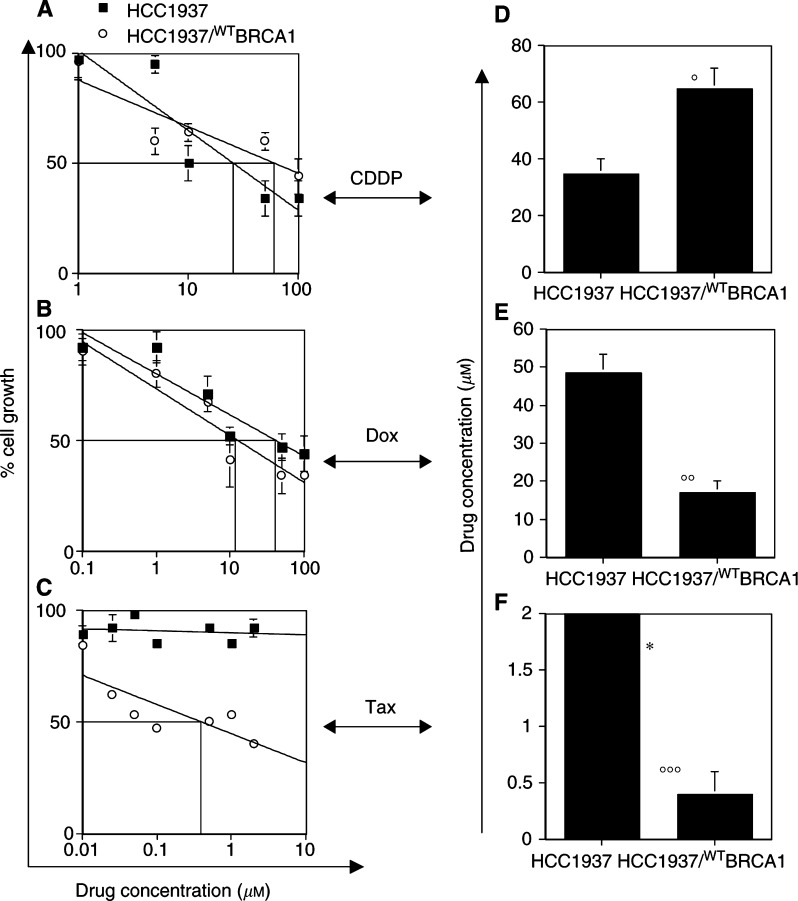
Full-length transfection of BRCA1 in HCC1937 (HCC1937/^WT^BRCA1) induces resistance to CDDP and restores sensitivity to Dox and Tax. In (**A**)–(**C**), interpolate logarithmic curves and the IC_50_ of cell growth after 48 h exposure to drugs are shown in each quadrant. In (**D**)–(**F**), the values of IC_50_ determined for both cell lines are shown. **°**: *P*<0.02; **°°**: *P*<0.05; **°°°**: *P*<0.001; ^*^: no IC_50_ was achieved at this drug concentration.

HCC1937 cells and the HCC1937/^WT^BRCA1 cells were analysed in parallel for drug-sensitivity assays. Figure 5A–C

shows that HCC1937/^WT^BRCA1 cells were less sensitive to the CDDP, and the IC_50_ concentration increased significantly (*P*<0.02) in HCC1937/^WT^BRCA1 cells, suggesting that BRCA1 may be involved in the repair process after the DNA damage induced by CDDP. On the other hand, reintroduced BRCA1 expression restored sensitivity to Dox and Tax. The IC_50_ concentration of Dox was significantly (*P*<0.05) lower in HCC1937/^WT^BRCA1 compared with the parental cells. HCC1937/^WT^BRCA1 cells treated with Tax also showed restored sensitivity to the drug. The IC_50_ in HCC1937/^WT^BRCA1 was in the range of 0.4–0.5 *μ*M, and the difference between these cells and the parental HCC1937 cells was significant (*P*<0.001).

### Effects of BRCA1 restoration on drug-induced apoptotic cell death

**Figure 6:**
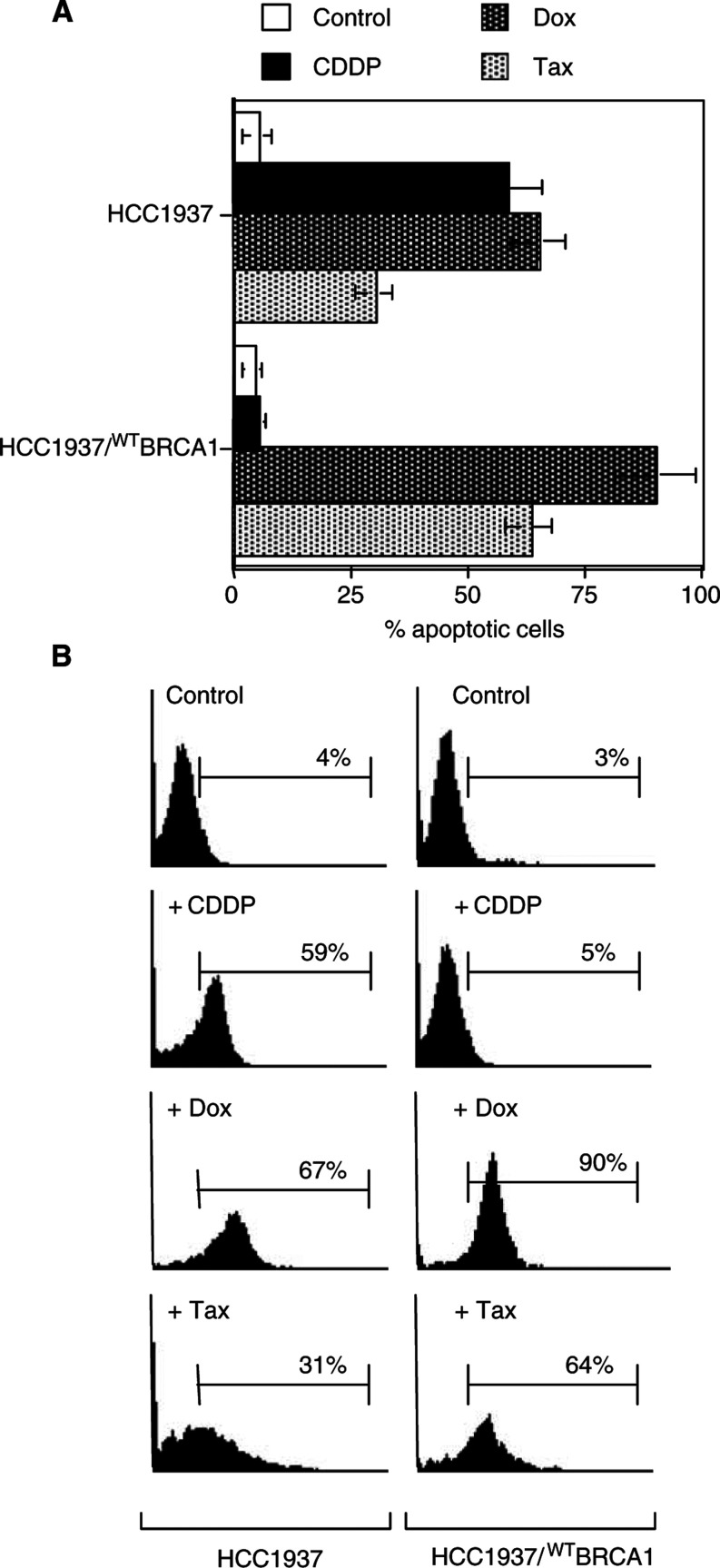
Effects of BRCA1-restored expression on drug-induced apoptotic cell death. In (**A**) percentages of apoptotic cells in untreated controls and after 48 h exposure to CDDP, Dox and Tax, respectively, for both BRCA1-defective and -competent cells are shown. In (**B**), flow cytometric profiles of Annexin-V FITC staining in a representative experiment are shown. Percentages of stained cells are reported in each quadrant.

In order to evaluate whether BRCA1 restoration might influence the apoptotic effects induced by cytotoxic drugs, we performed apoptotic assays on HCC1937 and HCC1937/^WT^BRCA1 cells (Figure 6

). Cells were treated for 48 h at the IC_50_-specific drug concentration, as measured for the most sensitive cell line, and apoptosis was detected by Annexin-V analysis. Induction of apoptotic cell death was detected in HCC1937 cells exposed to CDDP (30 *μ*M) and Dox (50 *μ*M). A weaker apoptotic effect was seen in HCC1937 exposed to Tax (2 *μ*M). Restoration of BRCA1 expression in HCC1937/^WT^BRCA1 cells resulted in a dramatic reduction of cell death induced by CDDP, whereas the majority of cells underwent apoptosis after exposure to Tax. A detectable increase in apoptotic events was also seen after exposure of HCC1937/^WT^BRCA1 cells to Dox. These data indicate that loss of BRCA1 function also influences the apoptotic response of breast cancer cells to cytotoxic drugs. However, flow cytometric analysis of Annexin-V detects early events in cells committed to programmed cell death, and therefore quantitative analysis of apoptosis is not precisely equivalent to the growth inhibition measured at a given time point.

## DISCUSSION

In this study, we have demontrated that BRCA1-defective breast cancer cells show a different sensitivity profile to anticancer drugs such as CDDP, Dox or Tax when compared with BRCA1-competent cells. These drugs are important cytotoxic agents for the therapy of breast cancer, both in the adjuvant setting and/or for the treatment of advanced disease. In our model, BRCA1-defective cells displayed a significant higher sensitivity to CDDP, whereas BRCA1 reconstitution significantly modified growth inhibition and apoptosis induction, supporting the idea that CDDP-induced DNA damage is strongly associated with the inactivation of BRCA1. These data are consistent with other observations demonstrating that inhibition of BRCA1 results in an increased sensitivity to CDDP (Lafarge *et al*, 2001). Even though the precise role of platinum compounds in the treatment of breast cancer remains to be defined, the interest for this class of drugs has been reawakened recently in the light of potential synergy with newer agents (Martin, 2001). CDDP is an active drug that can be safely and effectively combined with other recently introduced agents, such as vinorelbine, gemcitabine, Tax and docetaxel. Therefore, the possibility that hereditary breast tumours may specifically be more sensitive to this drug is highly suggestive.

The treatment of BRCA1-defective breast cancer cells with Dox or Tax *in vitro* showed an opposite response. Dox is a DNA helix intercalator and topoisomerase II inhibitor, and plays a fundamental role in the management of breast cancer, both as single agent as well as in combination with other drugs. Dox is considered the most active single agent in breast cancer. The potential resistance of hereditary breast tumors to Dox is an important issue considering the frequent use of this drug in the disease and the specific toxicity profile of anthracycline compounds. In fact, the clinical value of this drug is limited by late-onset ventricular dysfunction (Conte *et al*, 2000).

Recent reports in an experimental tumour cell system (Brodie *et al*, 2001) and also based on clinical observations (Kloos *et al*, 2001) appear to show conflicting results indicating that BRCA1 inactivation might indeed result in increased doxorubicin sensitivity. It has to be considered that the preclinical study involves BRCA1 conditional knockout mice, a clearly different situation as compared to human tumour cells, which might result from a different carcinogenetic process. The clinical study refers to doxorubicin-based combination chemotherapy, which also involves cyclophosphamide and 5 fluorouracil and it is therefore not possible to define the net role of doxorubicin in this setting. All these findings have, however, to be considered in the interpretation of our results. Lafarge *et al* have demonstrated that inhibition of BRCA1 led to enhanced sensitivity to etoposide that like Dox is a topoisomerase II inhibitor (Lafarge *et al*, 2001). This discrepancy could be because of the specific changes in other determinants of Dox and/or etoposide, that is, increased cellular drug clearance. Similar to Dox, the mitotic spindle poison Tax showed lower activity in BRCA1-defective cells when compared to BRCA1 wild-type cells, and the BRCA1 reconstitution dramatically reverted this specific profile, inducing sensitivity to Tax. These findings on Tax activity in BRCA1-defective breast cancer cells are consistent with other studies that have reported interference of BRCA1 expression with the antitumour effects induced by antimicrotubule agents in cells derived from sporadic breast tumours (Lafarge *et al*, 2001; Mullan *et al*, 2001). It is important to consider that Dox and Tax have been combined together in the treatment of breast cancer with high overall response rates (Sparano, 1999). However, unacceptable degrees of congestive heart failure has been reported in a subgroup of patients. In this context, it is of interest that BRCA1-defective cells *in vitro* are resistant to both drugs, suggesting that this treatment might be ineffective in breast hereditary cancer patients. It has been reported that anticancer drugs trigger apoptosis in various cancer cells, including solid tumours, suggesting that apoptotic events are associated with the antitumour activity of these drugs (Hickman, 1992; Seki *et al*, 2000). We have indeed found that the differences in cytotoxic activity were paralleled by differential induction of apoptosis.

In conclusion, our findings support the hypothesis of a specific pattern of chemosensitivity in BRCA1 breast hereditary carcinomas when compared with breast tumours arising in the sporadic setting. To our knowledge, our study is the first report where the effect of BRCA1 inactivation and reconstitution on drug sensitivity is evaluated in cells derived from a tumour arising in the BRCA1 hereditary setting. This appears to be of specific interest taking into account that these tumours appear to displace a peculiar gene expression profile as compared to BRCA2 and sporadic tumours (Hedenfalk *et al*, 2001). The results obtained in BRCA1 full-length transfected cells suggest that the BRCA1 gene product is a key modulator of drug sensitivity in breast cancer cells in this specifc setting and add novel information as compared to studies performed on breast sporadic tumours. These data therefore, support, the concept that inherited breast tumours are a different form of disease, suggesting the intringuing possibility of tailored treatment in this setting.
